# Combining Machine Learning, Single‐Cell Sequencing Data, and Mendelian Randomization Studies to Explore the Correlation Between Ischemic Stroke and Inflammatory Pathway Genes

**DOI:** 10.1155/ijog/7253270

**Published:** 2026-06-12

**Authors:** Si Wang, Yan Xu, Meilei Wang, Xinyi Zhang, Lin Ling

**Affiliations:** ^1^ Department of Cardiology, Xishan People′s Hospital of Wuxi City, Wuxi, Jiangsu, China; ^2^ Department of Neurology, Xishan People′s Hospital of Wuxi City, Wuxi, Jiangsu, China; ^3^ Department of Cardiology, The First Affiliated Hospital of Soochow University, Suzhou, Jiangsu, China, sdfyy.cn

**Keywords:** cytokine, inflammatory pathway, machine learning, Mendelian randomization study, scRNA-seq, tumor necrosis factor

## Abstract

**Background:**

Ischemic stroke (IS) is a severe neurological disorder, with inflammation playing a crucial role in its development. This study is aimed at investigating the gene expression profiles related to inflammation in IS patients and determining their association with disease progression.

**Methods:**

We analyzed two distinct gene expression datasets from public repositories to compare gene expression between IS patients and healthy controls. Key inflammatory pathway–related genes (IPRGs) associated with IS were identified through differential expression analysis and advanced machine learning techniques. Consensus clustering analysis was used to identify various inflammatory expression signatures in IS. Single‐cell sequencing was performed to dissect inflammation‐related signaling pathways. Additionally, Mendelian randomization studies were conducted to assess the causal relationship between tumor necrosis factor (TNF) and IS.

**Results:**

Four pivotal genes—HLA‐DRA, IL1A, IL15, and TNF—were found to be upregulated in IS patients and significantly correlated with inflammatory levels. A diagnostic model was developed and validated using a nomogram. Single‐cell sequencing analysis revealed variations in inflammatory pathway enrichment scores across different cell types, enhancing our understanding of immune cell infiltration patterns in IS patients and highlighting the critical roles of macrophages and monocytes in inflammation. Mendelian randomization studies suggested that TNF may have a negative regulatory effect on the risk of IS.

**Conclusion:**

This study provides insights into the gene expression profiles associated with inflammation in IS patients and identifies key IPRGs. These findings offer valuable information for understanding the pathogenesis of IS and emphasize the importance of inflammation in disease development. Our research also presents potential therapeutic targets and predictive tools for future stroke research and clinical practice.

## 1. Introduction

Ischemic stroke (IS) constitutes a considerable international health concern, profoundly undermining the health status and functional outcomes of populations across the globe [1]. The disorder typically arises from a vascular event that obstructs a cerebral artery, precipitating acute disruption of cerebral perfusion. This hemodynamic compromise can evolve into a critical neurological emergency with grave clinical implications [2]. The condition primarily stems from the occlusion of cerebral vessels, which induces localized ischemia and oxygen deprivation. This disruption in perfusion precipitates a cascade of metabolic failure and cellular injury within the affected brain territory, frequently culminating in devastating neurological outcomes [3]. Although advanced imaging techniques enable prompt identification of transient ischemic attacks (TIA), their practical utilization is often constrained by technical complexity and substantial expense. In contrast, circulating biomarkers reflecting oxidative stress, inflammatory activity, and neuronal injury represent a promising alternative for the diagnosis and prognostic assessment of IS. Such biomarkers provide a clinically attractive profile characterized by minimal invasiveness, rapid turnaround, and favorable cost efficiency [4].

Inflammation plays a central role in the pathological progression of IS. Following the initial ischemic insult, the resultant tissue injury initiates a robust inflammatory cascade, which in turn aggravates cerebral damage. This process involves the release of various inflammatory mediators, such as cytokines and other bioactive molecules, that drive a deleterious cascade contributing to secondary brain injury [5]. IS often disrupts the integrity of the blood–brain barrier (BBB). This breakdown facilitates the entry of peripheral inflammatory cells and neurotoxic compounds into the cerebral parenchyma, thereby exacerbating neural damage [6]. Moreover, the activation of immune cells including macrophages and astrocytes initiates the secretion of proinflammatory factors, which actively drive the neuroinflammatory cascade and contribute to subsequent tissue injury [7]. Therefore, managing the inflammatory response is a crucial strategy in IS treatment.

Inflammation and its associated cytokine networks are critically involved in the ischemic cascade. This neuroinflammatory response, driven by resident microglia and infiltrating peripheral immune cells, significantly contributes to neuronal and glial injury in the acute phase of cerebral ischemia.

[8]. In the early stages following ischemic injury, microglia undergo rapid phenotypic activation and secrete a spectrum of proinflammatory factors, such as interleukins, tumor necrosis factor (TNF), and chemokines. These factors, in turn, stimulate endothelial cells to enhance the expression of adhesion molecules, thereby promoting leukocyte adherence and intensifying the inflammatory cascade [9]. Inflammatory cues guide the transmigration of adaptive and innate immune populations—such as T lymphocytes, neutrophils, and monocytes—across the vasculature into the cerebral compartment. During the acute phase of IS, these infiltrating leukocytes serve as critical effectors of neuroinflammation and release key proinflammatory cytokines such as IL‐1*β*, IL‐6, and TNF‐*α* [10]. Overexpression of IL‐1*β* and TNF‐*α* exacerbates inflammatory responses and brain tissue damage [11, 12], whereas IL‐10, an anti‐inflammatory cytokine, protects brain tissues from further damage [13]. The multifaceted involvement of cytokines in IS implies that finely tuning their expression and bioactivity could contribute to attenuating inflammation‐driven injury and supporting neural repair and functional restoration. Further investigation remains necessary, however, to fully delineate the precise mechanisms through which individual cytokines influence stroke pathophysiology.

Although current research approaches still exhibit certain constraints, future investigations may further clarify the involvement of inflammatory processes and cytokines in IS. This may be realized by more precise dissection of underlying molecular pathways, advances in individualized therapeutic strategies, innovations in single‐cell profiling technologies, and rigorous assessment of causality. Together, these endeavors are expected to offer a more robust scientific basis for stroke prevention and treatment. In this work, through integrated analysis of transcriptomic and single‐cell sequencing datasets, we delineated the central involvement of inflammatory and immune activity in IS. By systematic profiling of inflammation‐associated genes, four central mediators—HLA‐DRA, IL1A, IL15, and TNF—were discerned as being strongly linked to the risk of IS. Furthermore, Mendelian randomization analyses indicated that TNF might exert a potentially causal influence on the development of this condition.

## 2. Materials and Methods

### 2.1. Sources and Preprocessing of Raw Data

Human peripheral blood transcriptomic profiles were sourced from two IS cohorts—GSE16561 (24 healthy controls and 39 IS patients) and GSE58294 (23 controls and 69 cardiogenic stroke cases)—retrieved from the Gene Expression Omnibus (GEO) repository [14]. In contrast, the GSE58294 cohort provided expression profiles for 69 cardiogenic stroke patients and 23 controls. Furthermore, the dataset′s clinical information notably contained detailed temporal records corresponding to the poststroke phase [15]. We standardized the initial microarray info via the “affy” suite. To bridge discrepancies across disparate platforms, interstudy variances were neutralized and the cohorts merged by leveraging the “sva” and “limma” computational libraries [16]. We acquired scRNA‐seq information regarding 58,528 cells from mouse brains (MCAO vs. sham; *n* = 3 per group) via the GSE174574 entry. After identifying 29 IPRGs within MSigDB, their collective transcriptional signatures across IS and CT subjects were evaluated by the “DESeq2” algorithm. To interpret these findings, visual plots were constructed by invoking the functions of “ggplot2” and “pheatmap” [17].

### 2.2. Machine Learning–Based Approach to Screen Hub Genes

The identification of key genes was achieved through a multimodel strategy involving Least Absolute Shrinkage and Selection Operator (LASSO), random forest, and support vector machine–recursive feature elimination (SVM‐RFE). Specifically, the random forest algorithm, implemented via the “randomForest” R package, evaluated variable significance. This supervised methodology calculated the average prediction error to assign an importance score to each candidate gene, enabling the selection of the top 10 IPRGs. In parallel, the SVM‐RFE algorithm was employed, chosen for its strong performance in datasets with a constrained sample size. This method systematically excludes irrelevant features while prioritizing those exhibiting high relevance to the outcome of interest. Feature selection via SVM‐RFE was further validated against a 10‐fold cross‐validation scheme to guarantee model stability and predictive accuracy [18]. To reduce the dimensionality of the feature space, we employed a regularized regression approach based on the L1 norm (commonly known as the LASSO). This technique is particularly effective when the number of predictors greatly exceeds the sample size. Using the “glmnet” library in R, we fitted a logistic regression model subject to L1 penalization. The optimal value of the regularization parameter (*λ*) was determined through 10 rounds of cross‐validation. This scheme enabled simultaneous feature selection and shrinkage of regression coefficients within the generalized linear modeling framework, thereby enhancing both predictive performance and model interpretability [19].

### 2.3. Construction and Validation of the Nomogram

The identified hub genes were integrated into a multivariate logistic regression framework. A nomogram was then generated using the “rms” R package. Model discrimination was measured by the area under the receiver operating characteristic curve (AUROC) via the “ROCR” toolkit. To evaluate prediction accuracy, predicted versus observed probabilities were compared using calibration plots. For clinical utility assessment, net benefit analysis (decision curve analysis) was carried out. Internal model stability was tested with 100 iterations of bootstrap resampling. The goodness‐of‐fit was formally quantified using the Hosmer–Lemeshow statistic along with corresponding calibration diagrams.

### 2.4. Consensus Cluster Analysis and Prediction of Immune Cell Infiltration

For unified data handling and graphical display of the CIBERSORT and MCPcounter outputs, the “IOBR” R package was employed. IOBR is a dedicated resource for multiomics immunoassays, encompassing eight previously published methods for accurately measuring the tumor microenvironment (TME) components in the context of cancer and other immunologic disorders [20]. Subtyping of the IS cohort was performed using the “ConsensusClusterPlus” R package. To identify the most robust cluster number, three criteria were applied: assessment of the rate of change in the consensus matrix, examination of the CDF curve visualization, and calculation of the CDF area under the curve (AUC). These steps facilitated the detailed comparison of clinical and pathological attributes among the resulting patient groups [21].

### 2.5. Processing of Single‐Cell Sequencing Data From Mouse Brain Tissue

Mouse brain scRNA‐seq data (GSE174574) were processed via the Seurat pipeline [22]. To guarantee the reliability of downstream analyses, rigorous quality filtering was applied. Cells exhibiting a mitochondrial read proportion (calculated via the “mitoRatio” approach) exceeding 10% were discarded, along with those having a total UMI count below 3. Additionally, cells that expressed fewer than 300 or more than 30,000 distinct transcripts were excluded. The remaining high‐quality cells underwent library size normalization using a log‐normalization routine (equivalent to the standard “LogNormalize” method). The normalized expression matrix was converted into a Seurat‐compatible object. Highly variable features were identified by selecting the top 2000 genes with the largest expression variance across cells. These features were then projected onto a reduced dimensional space via principal component analysis (PCA), retaining the first 20 components for subsequent clustering. A graph‐based approach leveraging the shared nearest neighbor (SNN) algorithm was employed to partition cells into clusters, and a two‐dimensional t‐SNE embedding was generated for visualization. Differentially expressed markers for each cluster were detected using a Wilcoxon rank‐sum test (the “FindMarkers” implement). Cluster identities were assigned by comparing their transcriptional profiles against a reference compendium using the SingleR methodology. Furthermore, a custom gene set enrichment score was calculated for each cell based on the expression of 29 inflammation‐related prognostic genes (IPRGs), employing the average expression scoring scheme available in the Seurat toolkit [23]. This approach first calculates the mean transcriptional level of all genes belonging to the target set, then divides the expression matrix into discrete bins according to those mean values, and finally randomly picks control genes from each bin to serve as a background reference. For monocytes, we subsequently reconstructed their developmental paths using the Monocle package, which orders cells along a pseudotime axis [24]. For trajectory inference, cell states were ordered along a pseudotemporal continuum by building a monocle object, reducing its dimensionality, and arranging cells according to developmental progression using standard settings. To explore cell–cell signaling, the CellChat toolbox was applied, leveraging network‐based algorithms to identify dominant outgoing and incoming communication patterns. Monocytes were first stratified by score into two subsets, after which CellChat was used to map intercellular communication networks, quantify ligand–receptor interactions, and deduce potential signaling events between cell groups [25].

### 2.6. Mendelian Stochastic Analysis

To evaluate causal effects of core genes on IS risk, we performed two‐sample MR analyses using genetic variants as proxies for gene expression. Causal estimates were derived through pleiotropy‐resistant approaches, including MR‐Egger and weighted median methods, to strengthen inference robustness. Summary statistics for exposures and outcomes were obtained from publicly accessible GWAS resources hosted by the IEU OpenGWAS initiative (https://gwas.mrcieu.ac.uk). Protein quantitative trait loci (pQTL) data for TNF, IL‐1*α*, and HLA‐DRA were acquired from a large‐scale plasma proteome atlas study [26]. Data on IS, on the other hand, were derived from a multidomain genome‐wide association study of 520,000 subjects [27]. All analyses were restricted to samples of European ancestry to minimize population stratification bias. The primary causal effects of core gene levels on IS risk were estimated via inverse‐variance weighted (IVW) regression, implemented within an R‐based Mendelian randomization framework. To evaluate potential pleiotropic bias, sensitivity analyses were conducted using MR‐Egger regression [28]. Manhattan plots were created using the “CMplot” R package.

### 2.7. Statistical Analysis

Statistical computations and data handling were carried out with R software (v4.2.1). For pairwise comparisons of continuous variables across two distinct cohorts, a rank‐based nonparametric test (Mann–Whitney *U*) was utilized. When comparing three or more independent groups, the Kruskal–Wallis rank‐sum test was adopted. To evaluate the relationship between two variables, the Spearman rank correlation coefficient was calculated. Throughout all analyses, a threshold of *p* < 0.05 was used to denote statistical significance.

## 3. Results

### 3.1. Identification of Hub Inflammatory Pathway–Related Genes in IS

**Figure 1:**
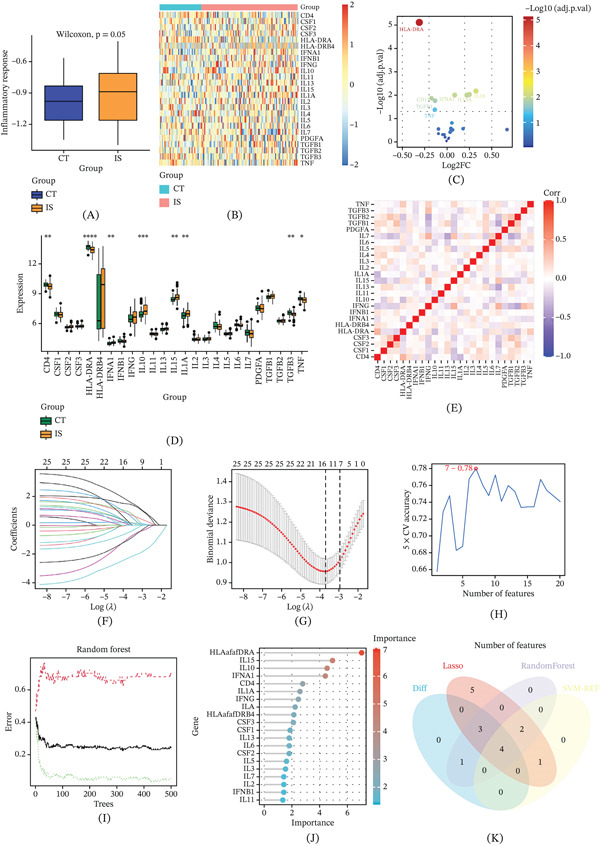
IPRGs in IS. (A) Box plots of inflammatory pathway enrichment scores evaluated by the ssGSEA method in normal controls (CT) and IS cases, with higher inflammatory response scores in the IS group. (B) Heat map illustrating the relative expression of IPRGs in CT and IS groups. (C, D) Volcano and box plots indicating that four IPRGs were upregulated and four were downregulated in the IS group. (E) The expression of IPRGs shows some correlation. (F) Tenfold cross‐validation for tuning parameter selection in the LASSO model, with each curve representing one gene. (G) LASSO coefficient analysis, with vertical dashed lines at the optimal lambda. (H) SVM‐RFE algorithm for selecting IPRGs with the highest classifier accuracy when the number of features is 7. (I) The relationship between the number of random forest trees and the error rate. (J) The ranking of the relative importance of IPRGs. (K) Venn diagrams showing the selection of four hub genes—HLA‐DRA, IL1A, IL15, and TNF—based on differential expression analysis, LASSO algorithm, random forest algorithm, and SVM‐RFE algorithm.

Gene expression data from the GSE16561 and GSE58294 cohorts were retrieved and processed. Normalization was performed using the “affy” package in R, and batch effects between the datasets were corrected via the “sva” package, resulting in a combined dataset of 47 controls and 108 IS samples (Figure S1A,B). Enrichment scores for the inflammatory pathway were calculated per sample using ssGSEA, revealing a pronounced inflammatory signature in the IS group compared with controls (Figure 1A). The expression patterns of IPRGs across groups are displayed in a heat map (Figure 1B). Differential expression analysis identified four upregulated and four downregulated IPRGs in IS (Figure 1C,D), and their correlative relationships are shown in Figure 1E. To pinpoint genes characteristic of IS, we applied three feature selection algorithms. LASSO regression selected 15 genetic markers (Figure 1F,G). SVM‐RFE yielded optimal classifier accuracy with seven features (Figure 1H), whereas random forest analysis stabilized at around 320 trees (Figure 1I). The top 10 IPRGs by importance were retained from random forest (Figure 1J). Integrating results from differential expression, LASSO, random forest, and SVM‐RFE identified four consensus IPRGs: HLA‐DRA, IL1A, IL15, and TNF‐*α* (Figure 1K).

### 3.2. Construction of the Nomogram and Analysis of Immune Infiltration

**Figure 2:**
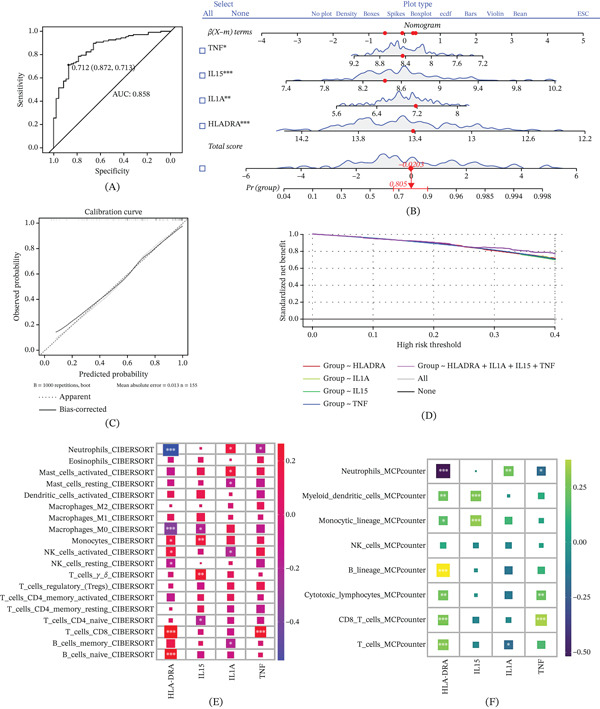
Analysis of nomogram construction and immune infiltration correlations. (A) ROC curves for IS diagnosis based on four hub genes. (B) Nomogram for OA diagnosis using four hub genes. (C) Calibration curve of the diagnostic model. (D) Decision curve analysis illustrating the clinical benefits of the nomogram. (E) Correlation between hub gene expression and 22 immune cell infiltrates (CIBERSORT). (F) Correlation between hub gene expression and the abundance of eight immune cells (MCP‐counter).

The combined four‐IPRG signature demonstrated a strong diagnostic performance, achieving an AUC of 0.858 in ROC analysis (Figure 2A). To translate this signature into a clinically usable tool, we constructed a risk prediction model (nomogram) incorporating these genes based on logistic regression (Figures 2B and S2A). This model assigns points for each gene, and the summed total points correspond to an individualized risk estimate for IS. The model′s predictive accuracy was confirmed by a well‐aligned calibration curve (Figure 2C) and its clinical utility was supported by decision curve analysis (Figure 2D). Internal validation via bootstrap resampling (100 replicates) yielded consistent performance metrics (AUC, specificity, sensitivity; Figure S2B–E). Furthermore, expression levels of these four IPRGs showed distinct correlations with immune cell infiltration profiles derived from CIBERSORT and MCP‐counter analyses (Figure 2E,F). Notably, TNF expression was positively associated with CD8^+^ T cell abundance in both algorithms.

### 3.3. Identification of Inflammation‐Related Clusters in IS

**Figure 3:**
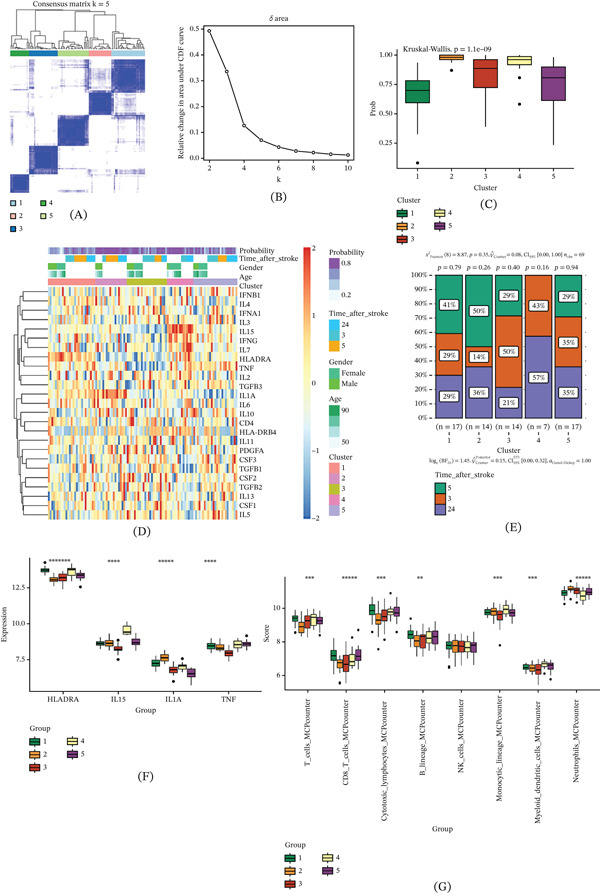
Clustering classification and identification of subgroup characteristics based on the four hub genes. (A) All IS cohort samples were classified into five clusters using a consensus clustering algorithm (k = 5). (B) Cumulative distribution function (CDF) for k values ranging from 2 to 10. (C) The probability of IS diagnosis for patients in the five clusters. (D) Expression heterogeneity of IPRGs in the five clusters was associated with various clinical traits. (E) Scale plot of the time since IS onset in patients across the five clusters. (F) Differential expression of the hub genes in the five clusters. (G) Differences in the abundance of eight types of immune‐related cells across the five clusters.

To delineate transcriptional subtypes associated with the four key IPRGs in IS, we performed consensus clustering of samples based on their expression profiles (Figure 3A). Evaluation of cumulative distribution function (CDF) curves supported K = 5 as the optimal cluster number (Figure 3B), leading to the stratification of the cohort into five distinct inflammation‐related subgroups. Clinical and molecular features—including gender, age, time from stroke onset, individual disease probability, and expression of all IPRGs—were integrated and visualized in a heat map, which revealed marked heterogeneity in the expression patterns of the four hub genes across clusters (Figure 3D). Notably, Cluster 1 exhibited the lowest estimated probability of stroke occurrence (Figure 3C). Although time since stroke onset did not differ significantly among the five clusters (Figure 3E), expression levels of the hub genes varied substantially between subgroups (Figure 3F). Immune deconvolution using the MCP‐counter algorithm indicated that Cluster 1, characterized by a lower disease likelihood, displayed elevated activity of T cells, B cells, and monocytes compared with other clusters (Figure 3G).

### 3.4. Identification of Inflammatory Patterns in IS Based on Single‐Cell Sequencing Data

**Figure 4:**
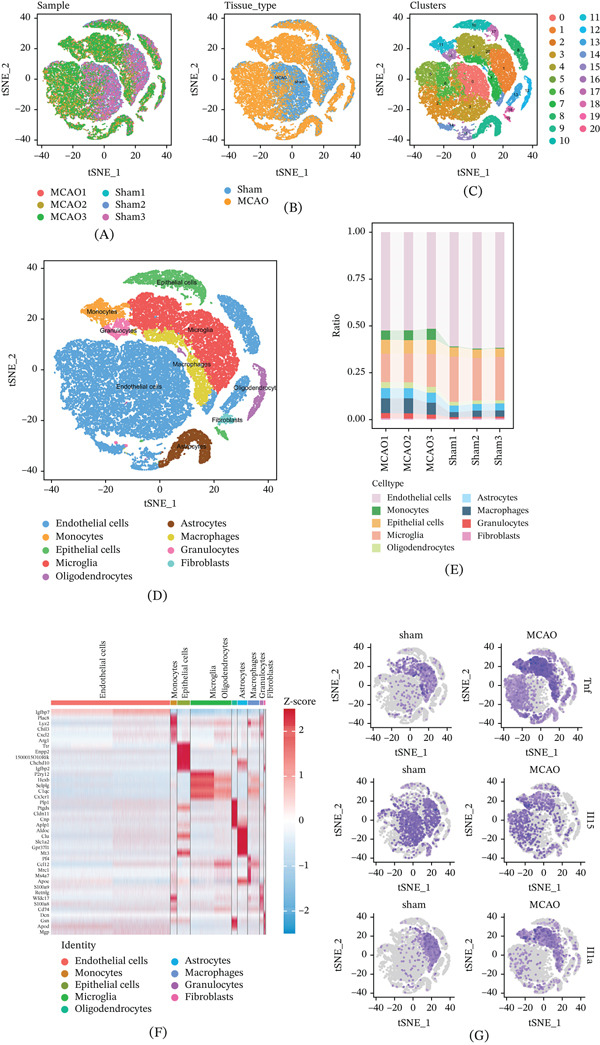
Classification of cell subpopulations in mouse brain tissue. (A–D) tSNE plots provide a visual representation of different samples, tissue sources, cell clusters, and cell subpopulations, each distinguished by different colors. (E) A histogram displays the distribution of cell types across various samples. (F) A heat map shows the relative expression levels of marker genes in eight distinct cell subpopulations. Genes with high expression are shown in red, whereas those with low expression are shown in blue. (G) The tSNE plot further illustrates the expression patterns of IPRGS in both sham and MCAO samples, facilitating a visual comparison of their expression profiles.

We acquired scRNA‐seq profiles from the GSE174574 dataset, comprising brain tissues from three mice subjected to middle cerebral artery occlusion (MCAO) and three sham controls. Following normalization for sequencing depth and gene counts, 2000 highly variable genes were selected and reduced via PCA. Subsequent clustering based on the top 20 principal components yielded 21 initial clusters, which were annotated into cell subtypes using the “SingleR” package. Visualization via t‐SNE effectively displayed sample origin, tissue region, cluster identity, and annotated cell populations (Figure 4A–D). Nine major cell types were identified, including fibroblasts, macrophages, endothelial cells, monocytes, epithelial cells, microglia, oligodendrocytes, astrocytes, and granulocytes. Endothelial cells constituted the predominant population across samples (Figure 4E). A heat map illustrates the top five marker genes per cell type (Figure 4F). Among the four core IPRGs, HLA‐DRA was not detected in this scRNA‐seq dataset; expression patterns of IL1A, IL15, and TNF in MCAO versus sham groups are visualized in t‐SNE space (Figure 4G).

**Figure 5:**
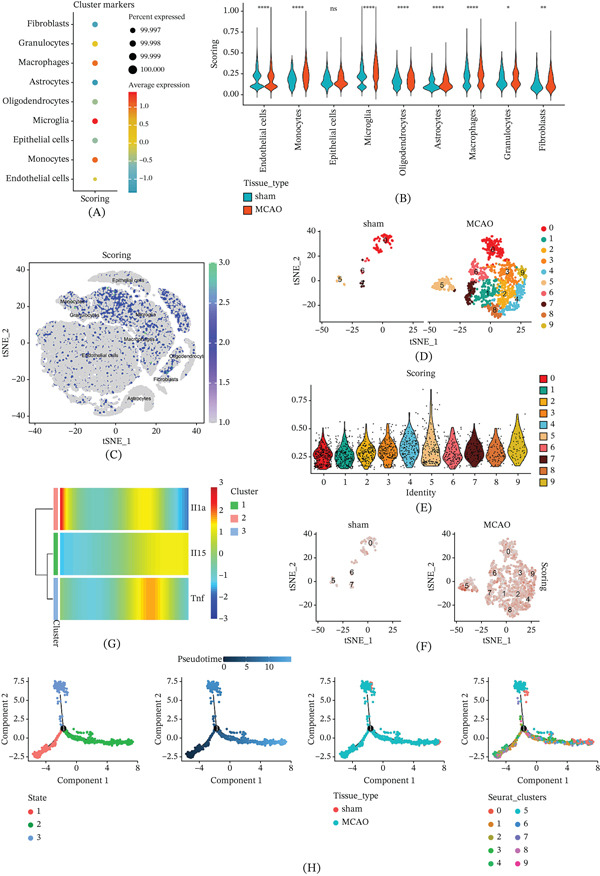
Enrichment scores of inflammatory pathways in cell subpopulations along with pseudotime analysis. (A) Bubble plots displayed the inflammatory pathway enrichment scores for each cell type in IS. (B) Differences in inflammatory pathway enrichment scores for each cell type between sham and MACO samples. (C) tSNE plots showed the inflammatory pathway enrichment scores for each cell type, where a darker green color indicated higher scores. (D) tSNE plots presented the re‐clustering of monocytes in sham and MACO samples, with a total of 10 monocyte subpopulations identified. (E) Differences in the inflammatory pathway enrichment scores of the 10 monocyte subpopulations. (F) tSNE plots displayed the inflammatory pathway enrichment scores of the 10 monocyte subpopulations. (G) A heat map illustrated the expression of four hub genes during monocyte development, with red representing high expression and blue representing low expression. (H) Cell trajectory and pseudotime analysis of monocytes was depicted.

Using the AddModuleScore algorithm, we quantified inflammatory pathway activity for each cell based on the expression of all 29 IPRGs. Monocytes, macrophages, and microglia exhibited notably higher enrichment scores (Figure 5A,C). Comparative analysis revealed elevated inflammatory scores across all cell types in MCAO samples relative to sham controls (Figure 5B). Given the observed prominence of monocytes in lower risk clusters, we isolated monocytes for subclustering, which defined 10 distinct subpopulations. Sham samples contained fewer monocytes, primarily residing in Clusters 0, 5, 6, and 7, whereas MCAO samples were enriched in other clusters (Figure 5D). Among these, Subcluster 5 demonstrated the highest inflammatory pathway score (Figure 5E,F). Pseudotemporal trajectory analysis reconstructed monocyte development, revealing three principal cell states along a continuum from sham‐ to MCAO‐associated phenotypes (Figure 5H). A heat map depicts dynamic expression of the four core IPRGs across this developmental continuum (Figure 5G).

**Figure 6:**
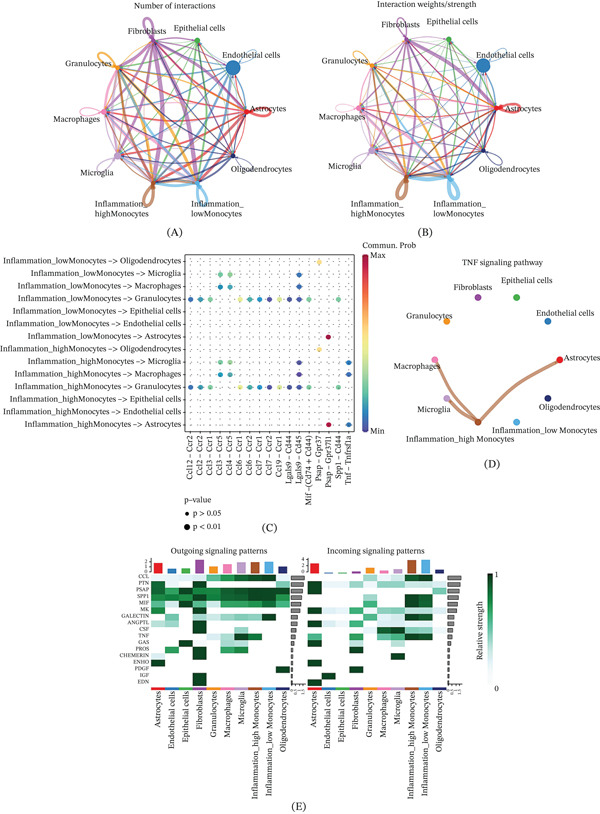
Analysis of cellular communication within inflammatory pathways. (A, B) The quantity of cell–cell interactions and overall interaction intensity were determined using the R software package “CellChat.” (C) Analysis of signaling pathways between various cell types. (D) Intensity of TNF signaling pathways among different cell types. (E) Heat map illustrating intercellular pathway analysis.

To decipher inflammation‐associated intercellular crosstalk in the ischemic brain, we performed cellular communication analysis using CellChat. Monocytes were stratified into high‐ and low‐score groups according to the median inflammatory pathway score. Network diagrams illustrated the quantity and strength of interactions between these monocyte subsets and other cell types (Figure 6A,B). Ligand‐receptor analysis highlighted the Tnf–Tnfrsf1a axis as a prominent signaling pathway linking high‐score monocytes with macrophages, microglia, and astrocytes (Figures 6C,D). Finally, we systematically evaluated outgoing and incoming signaling patterns across all cell populations (Figure 6E).

### 3.5. Two‐Sample Mendelian Randomization Analysis Suggests a Causal Relationship Between TNF and the Risk of Ischemic Stroke

**Figure 7:**
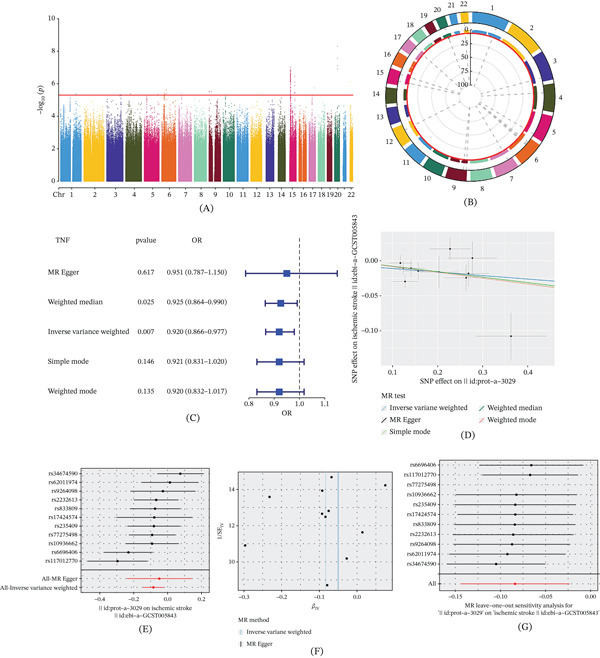
Outcomes of Mendelian randomization (MR) studies. (A, B) Manhattan plots illustrating the 11 selected SNPs. (C) Forest plot depicting the results from five MR methods. (D) Scatter plot showing the causal relationship between tumor necrosis factor and IS risk. (E) Forest plot displaying the causal effect of each SNP on IS risk. (F) Funnel plot illustrating the overall heterogeneity of the effect of tumor necrosis factor on IS. (G) Leave‐one‐out plot depicting the causal effect of tumor necrosis factor on IS risk.

The SNP profiles of TNF, IL1A, and HLA‐DRA associated with IS are provided in Table S1. For TNF, SNPs meeting genome‐wide significance (*p* < 5 × 10^−6^, *r* ^2^ < 0.001) were selected as instrumental variables (Figure 7A,B). MR analyses indicated a protective effect of TNF on IS risk. The primary IVW estimate yielded an OR of 0.92 (95% CI: 0.866–0.977, *p* = 0.007), which was supported by the weighted median method (OR 0.925, 95% CI: 0.864–0.990, *p* = 0.025). The MR‐Egger estimate was not statistically significant (OR 0.95, 95% CI: 0.787–1.150, *p* = 0.617) (Figure 7C). Scatter plots of the SNP–outcome associations illustrate the estimated causal effect across methods (Figure 7D), and forest plots detail the contribution of individual SNPs (Figure 7E). Sensitivity analyses showed no evidence of directional pleiotropy (MR‐Egger intercept *p* = 0.722) or substantial heterogeneity (Cochran′s Q *p* values > 0.05 for IVW and MR‐Egger) (Table S2). The funnel plot appeared symmetric (Figure 7F), and leave‐one‐out analysis confirmed that no single SNP drove the overall association (Figure 7G). Parallel MR analyses performed for IL1A and HLA‐DRA did not reveal significant associations with IS risk (Figure S3).

## 4. Discussion

IS, which accounts for nearly 87% of stroke incidents, typically results from the occlusion of cerebral arteries due to thrombi or atherosclerotic plaques. This vascular obstruction precipitates focal ischemia and hypoxia, culminating in neuronal injury and death. The predominant underlying pathology is atherosclerosis, a chronic inflammatory process driven by the deposition of lipid‐rich plaques within the arterial wall [29]. Clinically, reperfusion therapy via thrombolysis and/or endovascular procedures represents the cornerstone of acute IS management, aiming to rapidly re‐establish cerebral blood flow. However, its implementation faces significant challenges, including a stringent time frame for administration, inherent risks of adverse events, and inconsistent treatment responses. Consequently, only a limited subset of patients achieves timely access to these interventions [30]. Recent studies have highlighted the crucial role of inflammatory responses and interactions between the central nervous system and the peripheral immune system in the development of IS. Following cerebral ischemia, microvascular endothelial cells become activated, and the BBB is compromised, allowing inflammatory mediators to spread throughout the brain. Concurrently, numerous activated glial cells and peripheral immune cells infiltrate the infarcted areas, leading to a systemic inflammatory state [31]. Thus, targeting the immune‐mediated inflammatory response is a critical goal in stroke treatment. However, numerous immunomodulators that are aimed at both innate and adaptive immunity in IS have yet to meet expectations in clinical trials, highlighting significant translational challenges [32].

Recent years have seen an increasing application of MR methodology in unraveling the pathogenic mechanisms of immune‐related diseases. In the field of cerebrovascular disorders, for instance, MR analysis by Mo et al. demonstrated a causal link between neutrophil activation and intracerebral hemorrhage [33]. Regarding autoimmune diseases, bidirectional MR investigations have unraveled the intricate interaction networks between thyroid dysfunction and myasthenia gravis [34]. Particularly noteworthy is that the immunocyte phenotyping approach analogous to our study design has been successfully implemented in mechanistic studies of various conditions, including biliary tract cancer and pancreatitis, thereby providing cross‐disease validation for our findings [35–37]. Collectively, these studies suggest that immune dysregulation may represent a common pathological substrate across multiple disease systems.

Inflammatory mediators are pivotal in the pathogenesis of IS. Following the onset of ischemia, a swift inflammatory signaling cascade is triggered, leading to the release of multiple regulatory proteins. Key proinflammatory cytokines, including TNF, IL‐1, and IL‐6, are predominantly secreted by activated immune cells. Experimental models of stroke indicate that the systemic inflammatory response is characterized by a rapid elevation of serum cytokine concentrations within hours postevent, concurrent with heightened production of inflammatory factors by circulating and splenic immune populations—a decisive stage in the IS‐associated inflammatory cascade [38]. These cytokines exert dual effects in IS pathogenesis. While they modulate inflammation by recruiting and activating immune cells—potentially exacerbating tissue injury—they also contribute to processes involved in cerebral repair and regeneration [39]. The mechanisms of inflammatory cytokines in IS are multifaceted. They influence brain tissue injury and repair through various pathways. For instance, they can increase vascular permeability, causing brain edema and BBB disruption. They can also activate neurons and glial cells, leading to apoptosis and inflammation. Additionally, these signaling molecules stimulate neural stem cell proliferation and differentiation, thereby supporting the repair and regeneration of brain tissue [40]. Research indicates that inhibiting the production and activity of inflammatory cytokines could be advantageous in treating IS. Certain drugs can suppress the synthesis of these cytokines or block their receptor signaling, thereby reducing damage to brain tissue [41]. In conclusion, inflammatory cytokines are crucial in IS. Further research will enhance our understanding of the inflammatory response mechanisms in IS and offer new targets and strategies for treatment.

This paper seeks to enhance our understanding of the inflammatory immune response mechanisms in IS and to inform the development of drugs targeting inflammatory damage for IS treatment. We identified 29 IS‐related IPRGs from public gene expression datasets and pinpointed four key IPRGs: HLA‐DRA, IL1A, IL15, and TNF using machine learning algorithms. HLA‐DRA, a member of the human leukocyte antigen (HLA) family, is crucial for immune system function. It is believed to modulate the immune response in stroke by influencing the activation and function of immune cells, thereby affecting stroke progression and outcomes [42]. IL1A is a proinflammatory cytokine that plays a significant role in stroke. Its mechanisms in IS are diverse. IL1A can increase the expression of cytokines, chemokines, and growth factors, activate matrix metalloproteinases, upregulate adhesion molecules, enhance leukocyte infiltration, activate platelets, alter blood flow, promote angiogenesis, and inhibit neurogenesis [8]. IL‐1A enhances astrocyte‐derived nerve growth factor production, driving astrocytic proliferation and glial scar formation to attenuate ischemic damage [43]. IL‐1A can also aggravate ischemic damage by targeting endothelial cells, elevating adhesion molecule expression, and consequently compromising the integrity of the BBB [44].

Interleukin‐15 (IL‐15) is implicated in stroke pathology. Synthesized by activated monocytes/macrophages, epithelial cells, and fibroblasts, IL‐15 shares structural and functional homology with interleukin‐2 (IL‐2). It mediates chemotaxis, promotes cell survival, and participates in neuroinflammatory processes, underscoring its multifaceted role in the pathophysiology of stroke [45]. IL‐15 serves as a potent T‐cell chemoattractant, facilitating their infiltration into inflammatory foci. Emerging evidence points to a pivotal role for astrocytes, which are now known to secrete inflammatory cytokines such as IL‐15, thereby shaping cell‐mediated immunity in stroke. This astrocyte‐driven inflammatory milieu promotes the accumulation of CD8^+^ T cells and natural killer (NK) cells, both of which are critically involved in the pathogenesis of cerebral ischemic injury. This underscores the intricate cytokine‐mediated crosstalk between immune activation and stroke progression [46]. Recent studies have also reported that IL‐15 modulates cortical neuronal responses to ischemia by attenuating endoplasmic reticulum stress and increasing cell survival [47]. Finally, TNF, a significant proinflammatory cytokine encoded by the TNF gene, participates in regulating inflammation, apoptosis, and the activation of immune cells.

These genes are abnormally expressed in patients with IS and can effectively predict its onset. Machine learning can identify biomarkers related to stroke development and progression, aiding in monitoring patient conditions, treatment responses, and predicting future risks. Researchers have made initial progress in validating biomarkers associated with IS [32]. However, stroke represents a disease of complex etiology involving interplay between numerous genetic and environmental influences; thus, relying solely on individual genetic markers presents challenges in fully elucidating its pathogenic mechanisms.

Based on the expression profiles of four key genes, samples were stratified into five inflammatory subtypes. The cluster associated with minimal stroke risk displayed elevated activity of T‐cells, B‐cells, and monocytes, implying a potential protective effect in IS. Single‐cell analysis in an IS mouse model further pinpointed monocytes, macrophages, and microglia as central contributors to the inflammatory cascade. Interactions among these cells appear to be regulated significantly by the Tnf–Tnfrsf1a axis, which helps modulate immune homeostasis by constraining excessive inflammation while promoting anti‐inflammatory mechanisms [48]. Following stroke, these cells rapidly accumulate within the injured brain tissue, where they release anti‐inflammatory cytokines such as IL‐10 to suppress inflammation and promote immune tolerance [49]. These cells modulate inflammatory processes through the secretion of mediators including cytokines and chemokines, which orchestrate the migration and functional activation of other immune cells.

## 5. Conclusion

In conclusion, our study confirmed the causal relationship between TNF and IS risk through a two‐sample Mendelian randomization analysis, suggesting a theoretical basis for TNF as a therapeutic target for IS. However, OR from this analysis was less than 1, indicating that TNF may act as a protective factor against IS. Although TNF‐*α* is traditionally viewed as a proinflammatory cytokine that mediates inflammation in IS, its overexpression can lead to increased inflammation and neuronal cell death [50]. Research indicates that TNF‐*α* triggers vascular endothelial cells to upregulate adhesion molecules, facilitating the adhesion and transmigration of inflammatory cells. This process contributes to cerebrovascular inflammation and the impairment of endothelial function [51]. Moreover, TNF‐*α* can activate neurons and glial cells, potentially causing neuronal damage and neuroinflammation. However, moderate levels of TNF‐*α* may actually protect against stroke. This is because moderate TNF‐*α* expression aids in clearing and repairing the inflammatory response, thereby helping to restore brain tissue function. Additionally, TNF‐*α* may enhance brain tissue repair and regeneration by promoting neurogenesis and synaptic plasticity [52]. In summary, while overexpression of TNF may elevate the risk of IS, moderate levels of TNF‐*α* could potentially offer protection against IS. TNF′s specific mechanisms and functional impact in IS progression require further elucidation.

Our study offers several innovations and advantages. Firstly, we employed multiple machine learning algorithms, coupled with differential expression and survival analyses, to identify four key genes from 29 candidate IPRGs. This approach enhances the objectivity and accuracy of selecting feature genes. Based on the four key genes, we constructed a risk‐prediction nomogram, providing a straightforward and effective tool for IS risk assessment and clinical decision support. Secondly, using single‐cell sequencing data, we revealed the heterogeneity and dynamic changes of different cell types and signaling pathways in an IS model, offering novel insights into the IS immune microenvironment. Finally, a two‐sample Mendelian randomization analysis established a causal relationship between TNF and IS risk, laying a foundation for further investigation into TNF’s role in IS pathogenesis. Furthermore, in accordance with our review of the relevant study by Hu Y. et al. [53] and the study by Zhao S. et al. [54], we have formally cited these works in our manuscript to adhere to academic standards and preclude potential copyright concerns.

However, the present investigation is constrained by its reliance on existing omics repositories and a relatively modest sample cohort, which may affect the robustness and broader applicability of the conclusions. Future research would benefit from validating these findings in larger, multicenter cohorts or through integrated analysis of independent datasets. Moreover, while comprehensive in silico analyses were performed, the study lacks direct functional validation of the prioritized mechanisms. Subsequent mechanistic studies, particularly those employing single‐cell sequencing and functional assays, are essential to experimentally confirm the biological and pathological relevance of the identified cellular dynamics and pathway alterations.

## Author Contributions

**Si Wang:** Writing—conceptualization, data curation, methodology, software, visualization, writing—original draft. **Yan Xu:** formal analysis, methodology, software, visualization, writing—original draft. **Meilei Wang:** project administration, supervision, writing–review and editing. **Xinyi Zhang:** investigation, writing—original draft. **Lin Ling:** writing—funding acquisition, supervision, validation, writing—review and editing. **Si Wang, Yan Xu, Meilei Wang, and Xinyi Zhang** contributed equally to this work.

## Funding

This study was supported by the Research Project of Soochow University (Nos. H230885 and H220142).

## Ethics Statement

This article does not contain any studies with human participants or animals.

## Conflicts of Interest

The authors declare no conflicts of interest.

## Supporting Information

Additional supporting information can be found online in the Supporting Information section.

## Supporting information


**Supporting Information 1** Figure S1: PCA plots of the GSE16561 and GSE58294 cohorts before and after removal of the bulk effect.

**Supporting Information 2** Figure S2: (A) OR values and confidence intervals for hub genes; (B) validation of ROC curves by replicate sampling; (C) range of distribution of area under AUC; (D) range of distribution of sensitivity; and (E) range of distribution of specificity.

**Supporting Information 3** Figure S3: (A) Forest plot of the causal effect of SNP for interleukin‐1*α* on IS risk. (B) Forest plot of the causal effect of SNP for human leukocyte antigen‐DR*α* on the risk of IS.

**Supporting Information 4** Table S1: SNP information for TNF, IL1A, and HLADRA in IS.

**Supporting Information 5** Table S2: Analysis of heterogeneity and horizontal polytomousness.

## Data Availability

The transcriptomic datasets (GSE16561 and GSE58294) and single‐cell RNA sequencing data (GSE174574) analyzed in this study are available in the Gene Expression Omnibus (GEO) repository (https://www.ncbi.nlm.nih.gov/geo). The Mendelian randomization summary statistics were obtained from the IEU OpenGWAS database (https://gwas.mrcieu.ac.uk) and relevant published pQTL and GWAS studies cited within the manuscript. All codes and intermediate data generated during the study are available from the corresponding author upon reasonable request.
