# Supplementary figures and images for: Combining Machine Learning, Single‐Cell Sequencing Data, and Mendelian Randomization Studies to Explore the Correlation Between Ischemic Stroke and Inflammatory Pathway Genes
Source: Int J Genomics. 2026 Jun 12;2026:7253270. doi: 10.1155/ijog/7253270 (PMC13261689; doi:10.1155/ijog/7253270)

**A**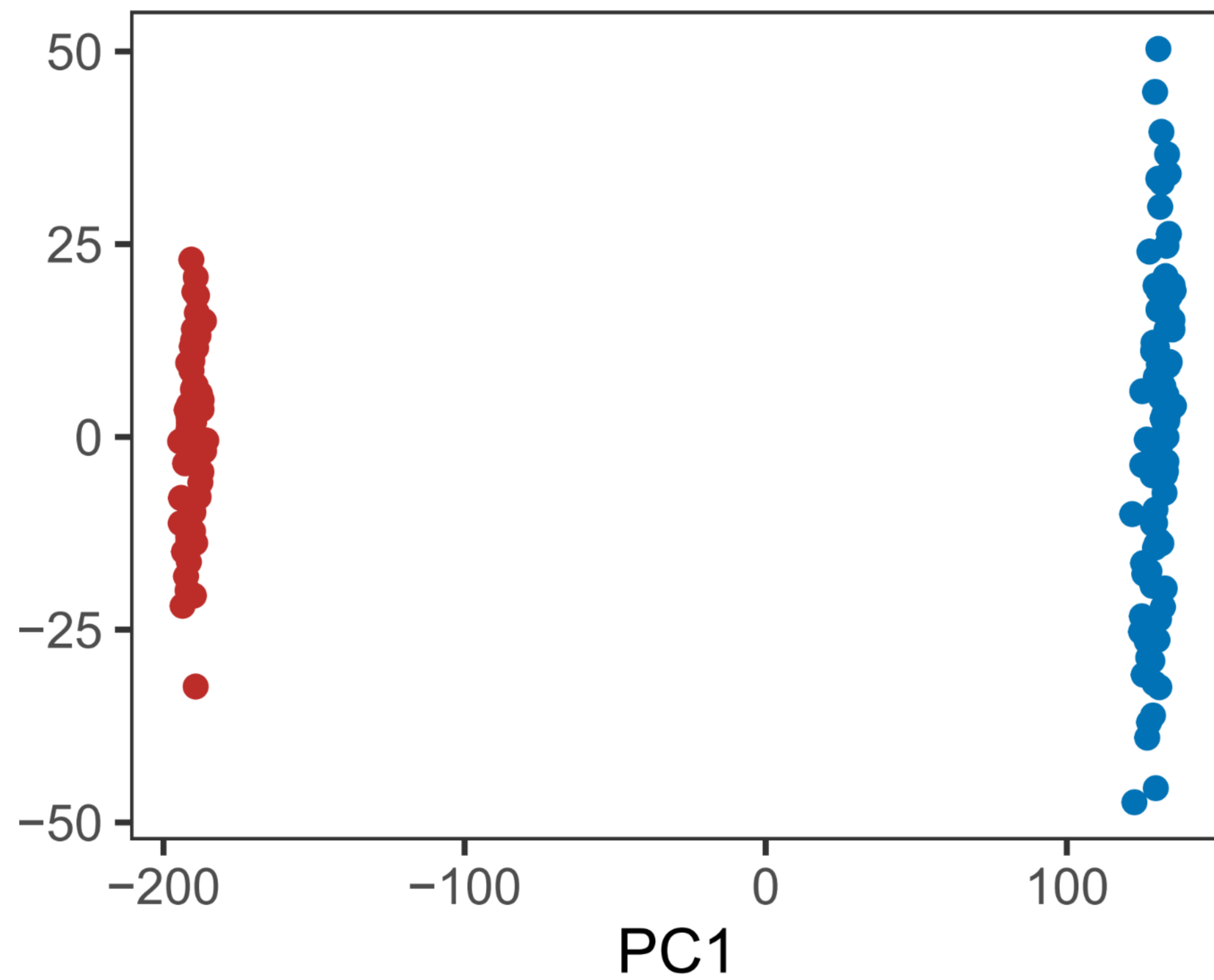**B**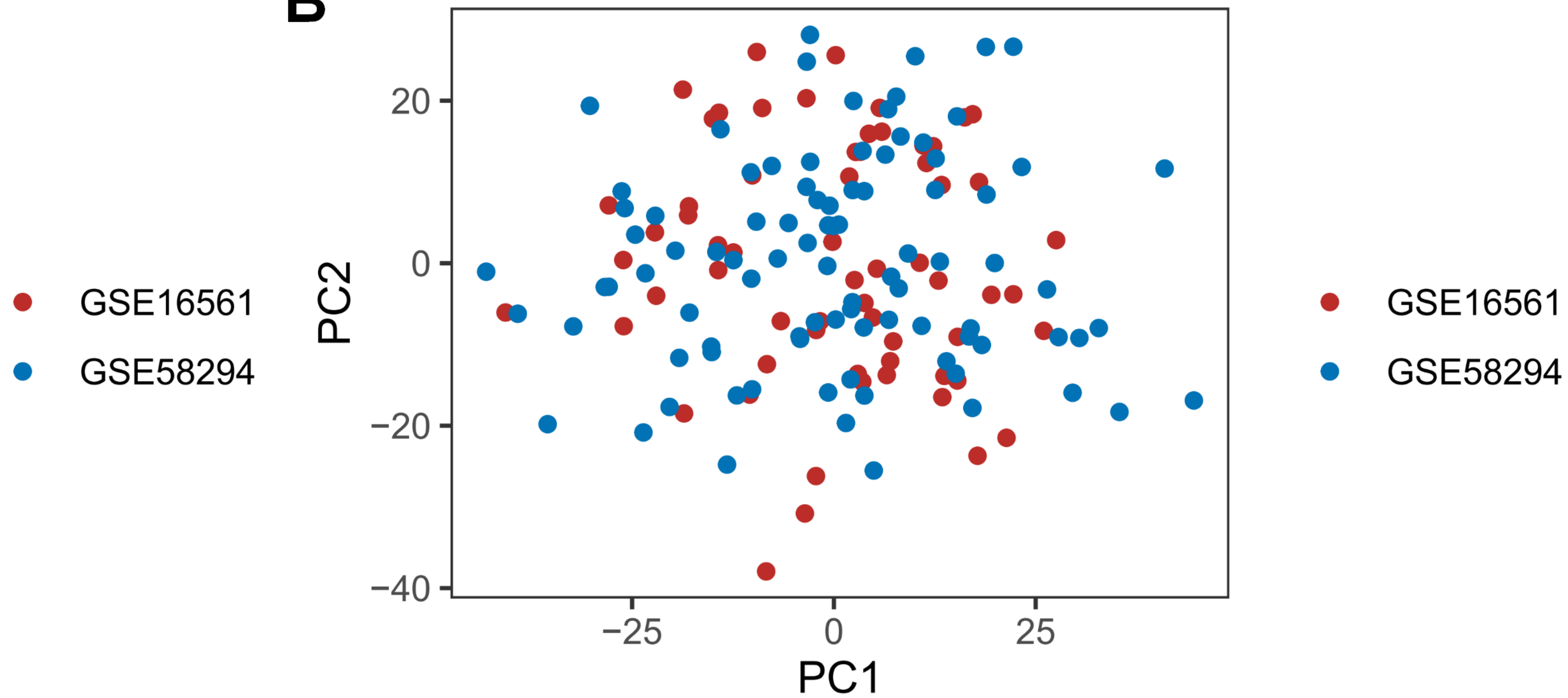

Supplement: Supplementary file 1 — Supporting Information 1 Figure S1: PCA plots of the GSE16561 and GSE58294 cohorts before and after removal of the bulk effect. [file IJOG-2026-7253270-s001.pdf]

**A**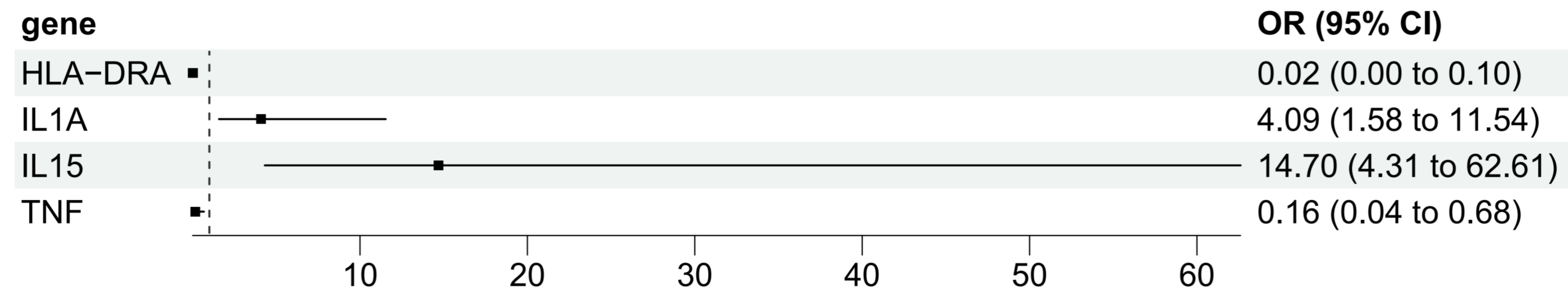**B**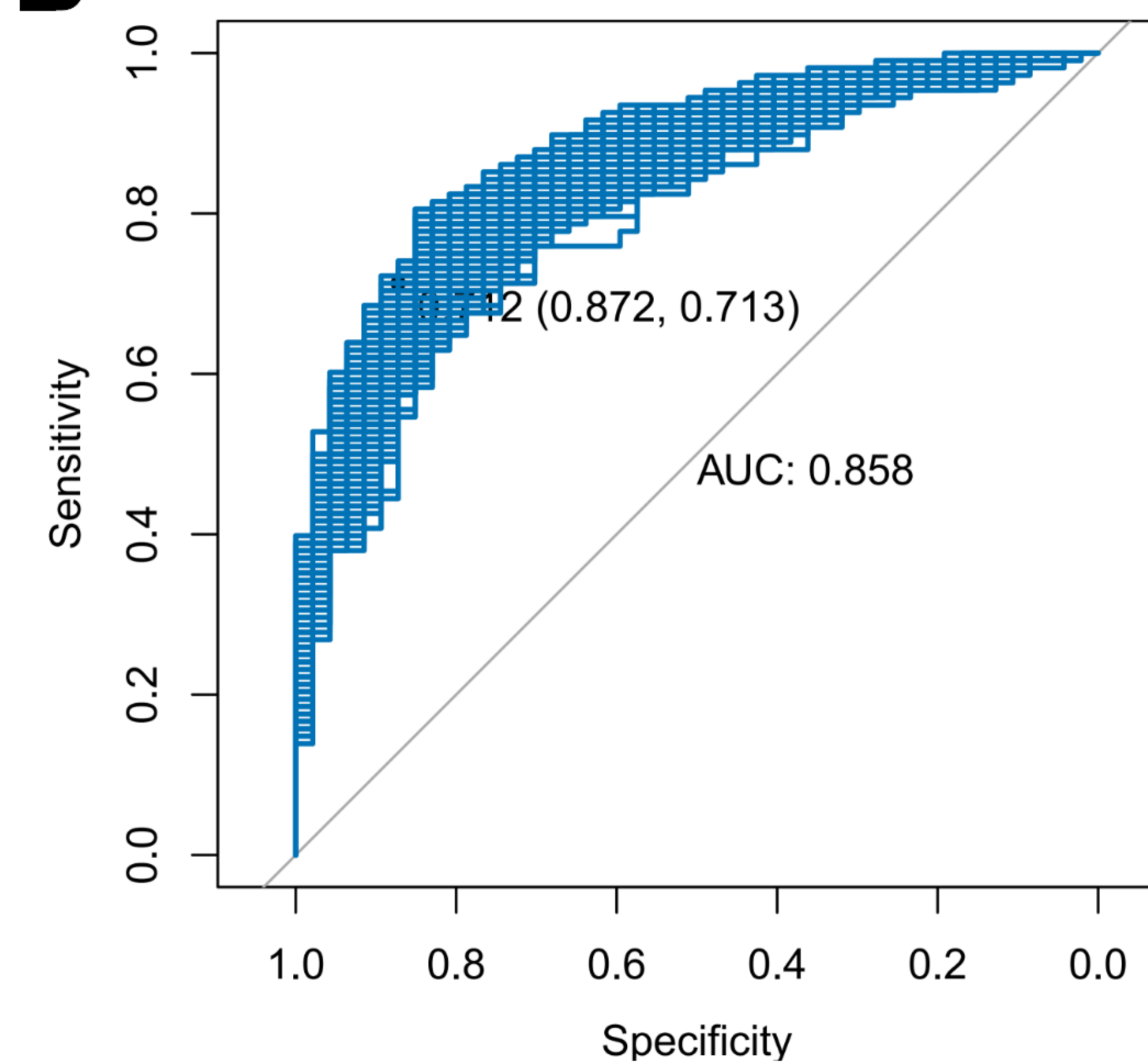**C****Histogram of  $t$** 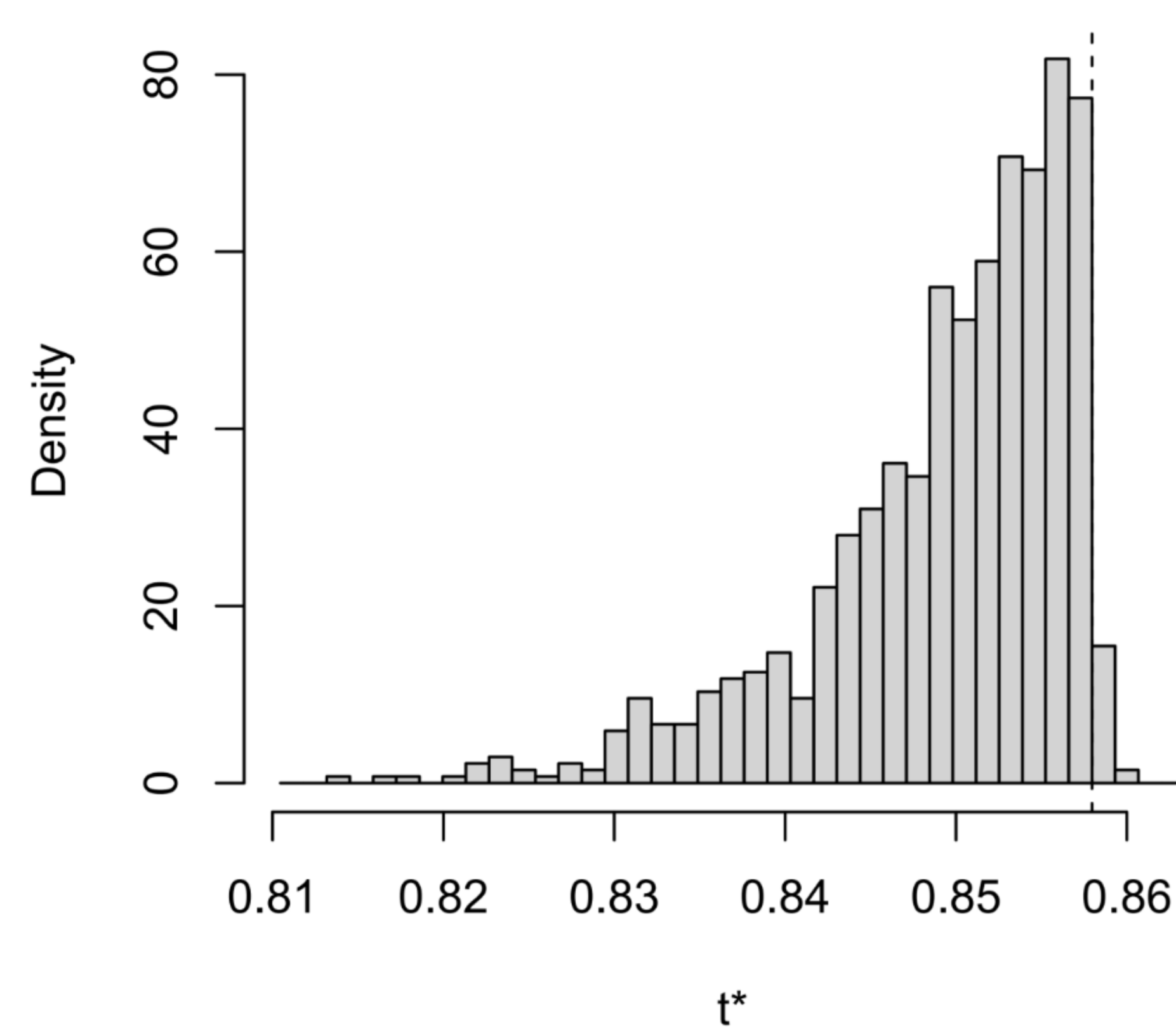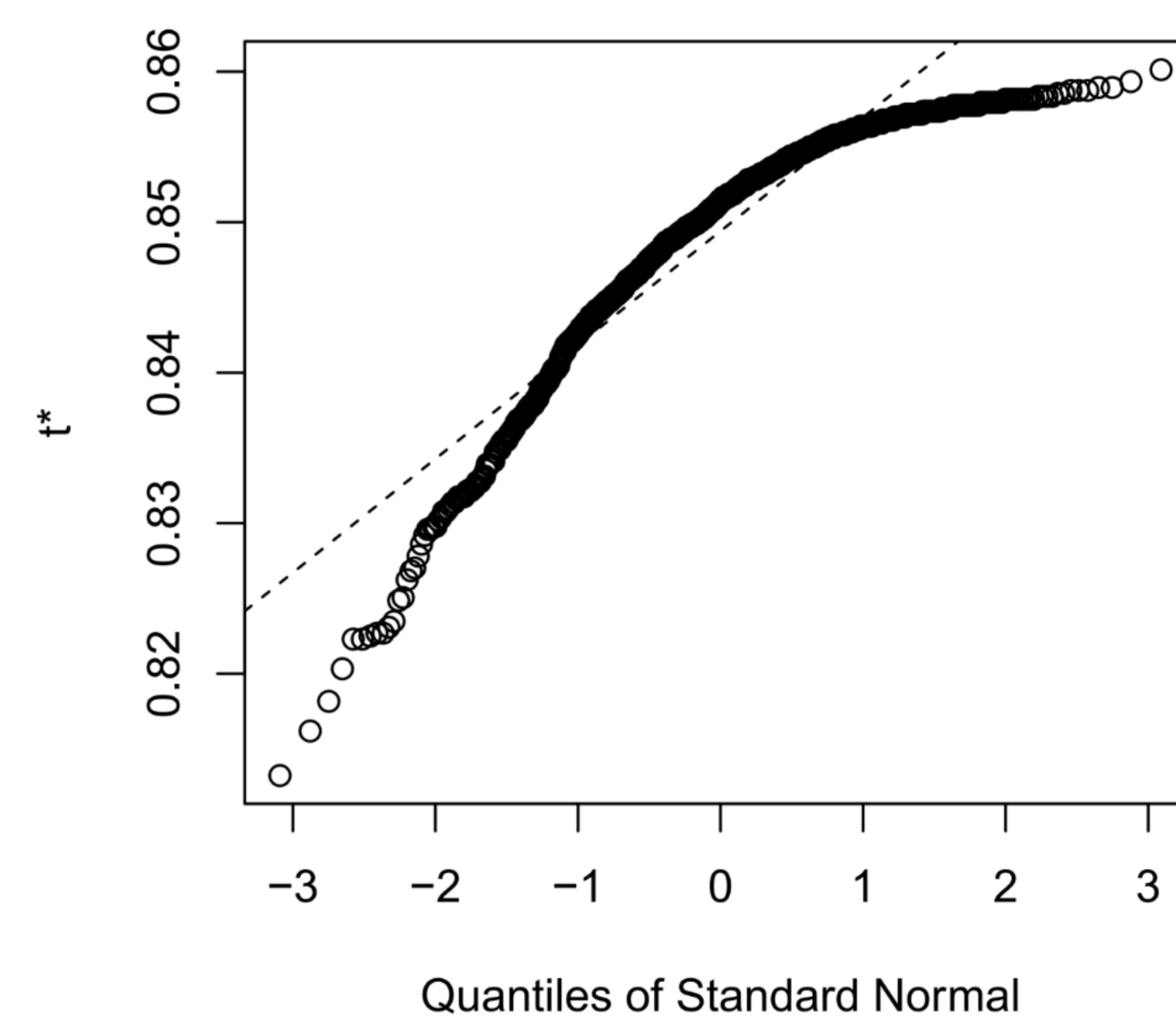**D****Histogram of  $t$** 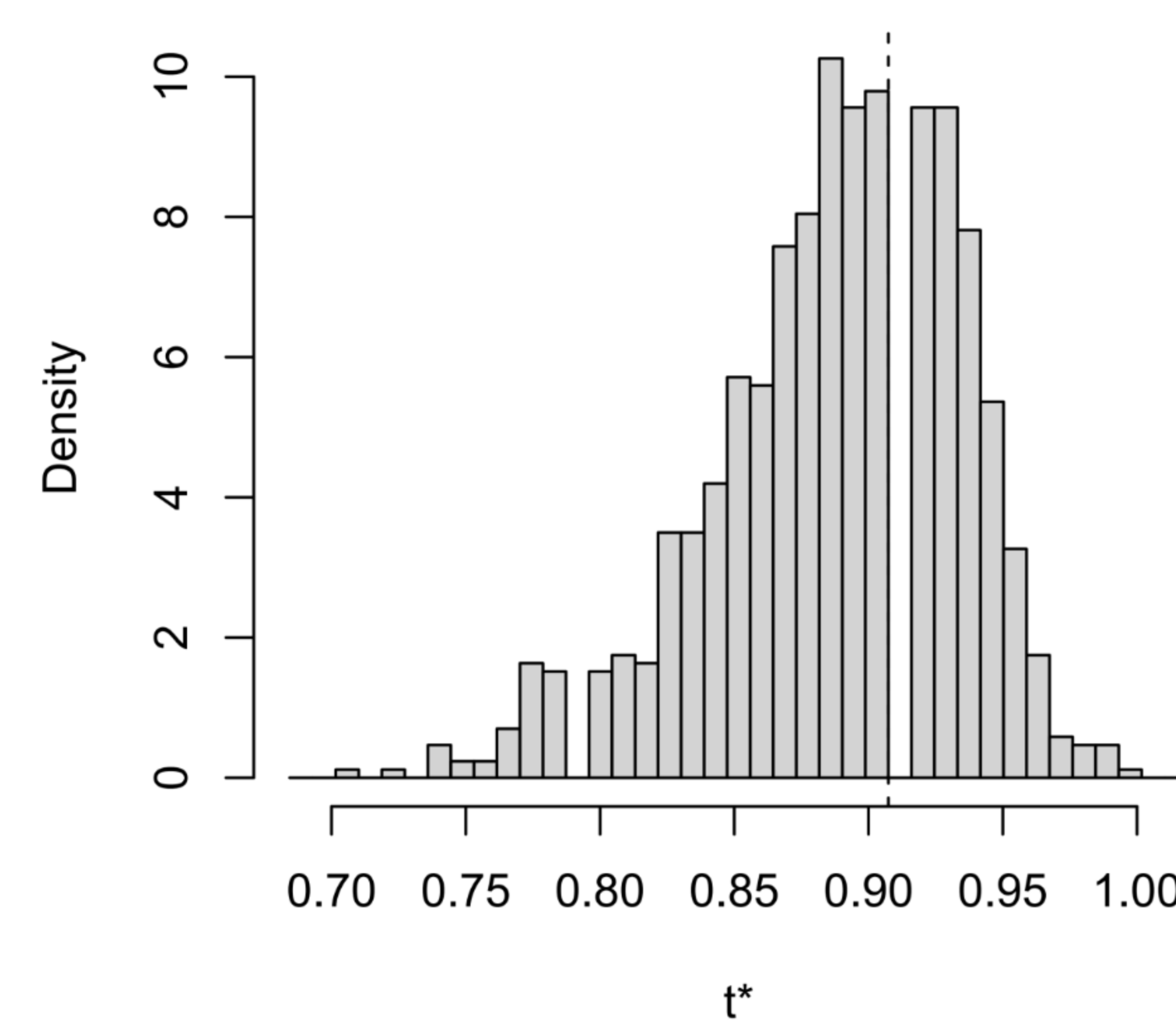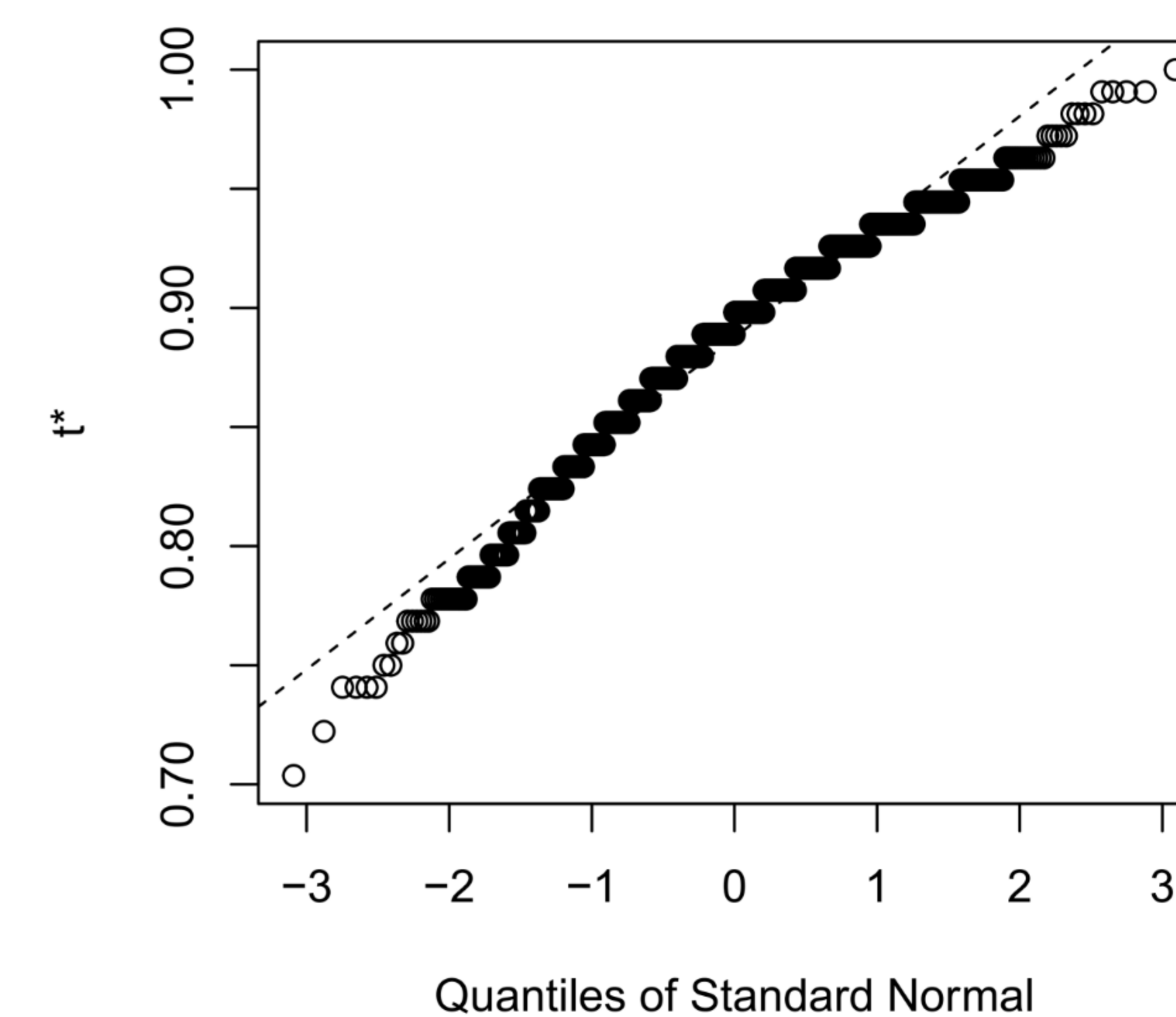**E****Histogram of  $t$** 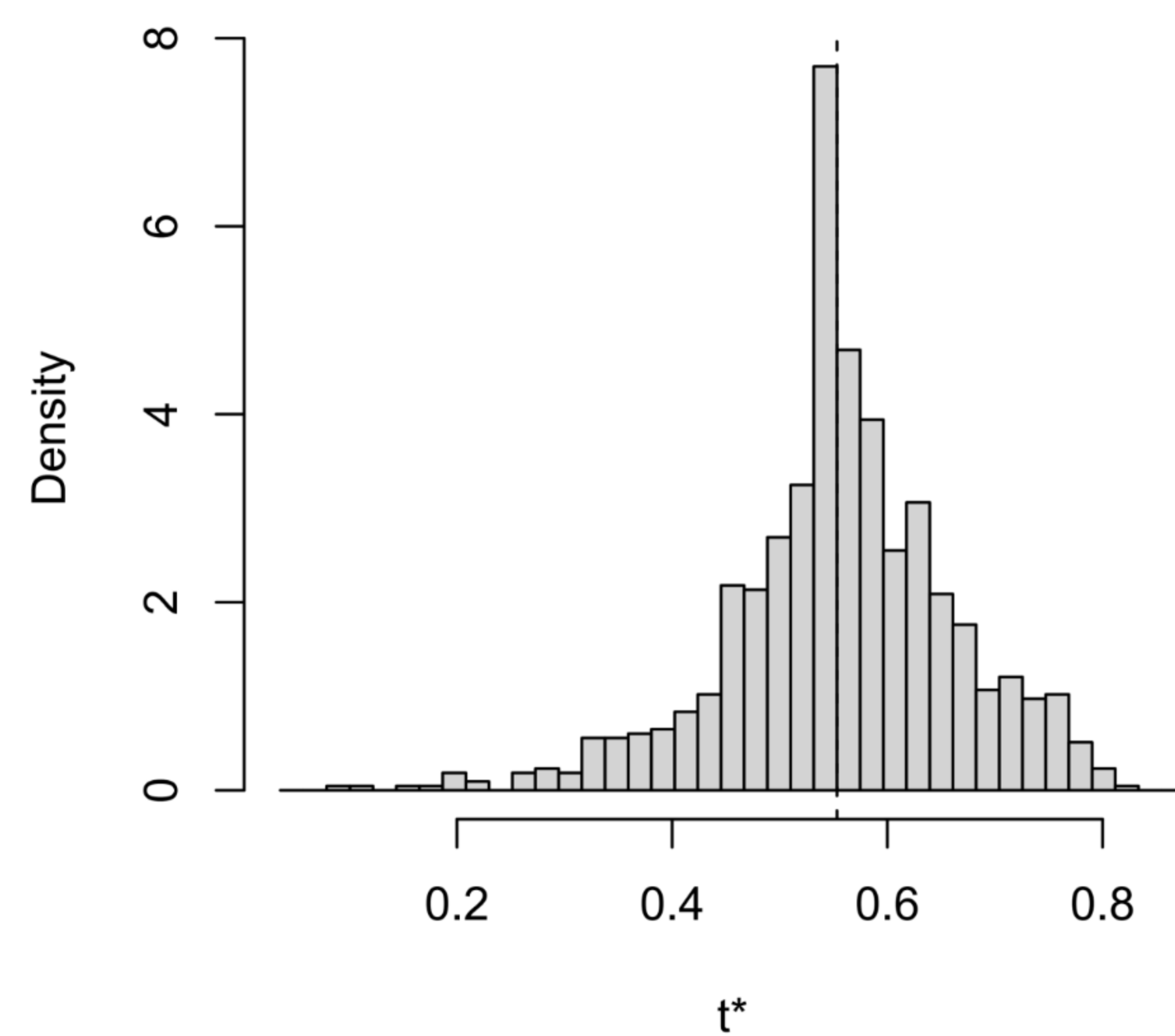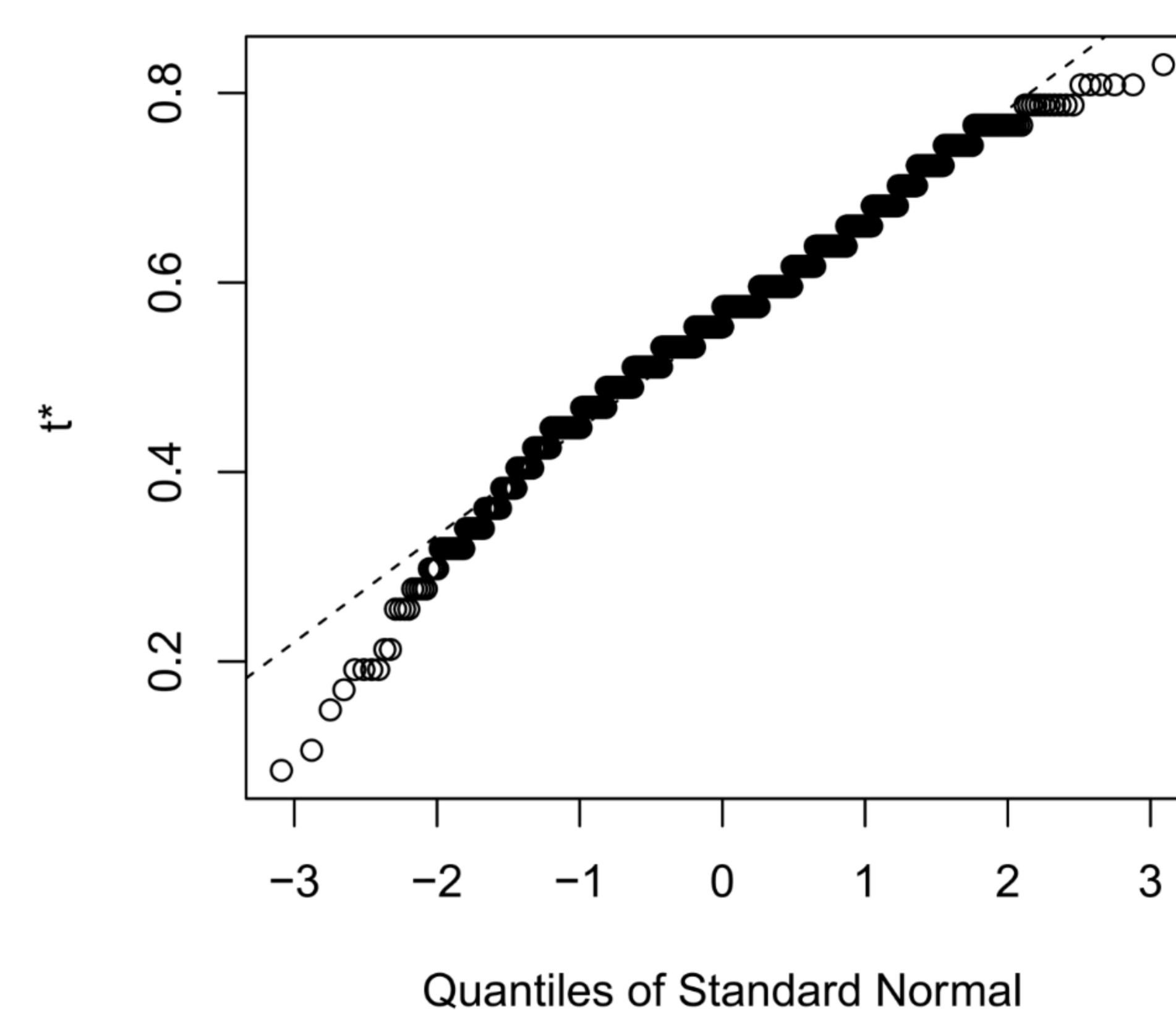

Supplement: Supplementary file 2 — Supporting Information 2 Figure S2: (A) OR values and confidence intervals for hub genes; (B) validation of ROC curves by replicate sampling; (C) range of distribution of area under AUC; (D) range of distribution of sensitivity; and (E) range of distribution of specificity. [file IJOG-2026-7253270-s002.pdf]

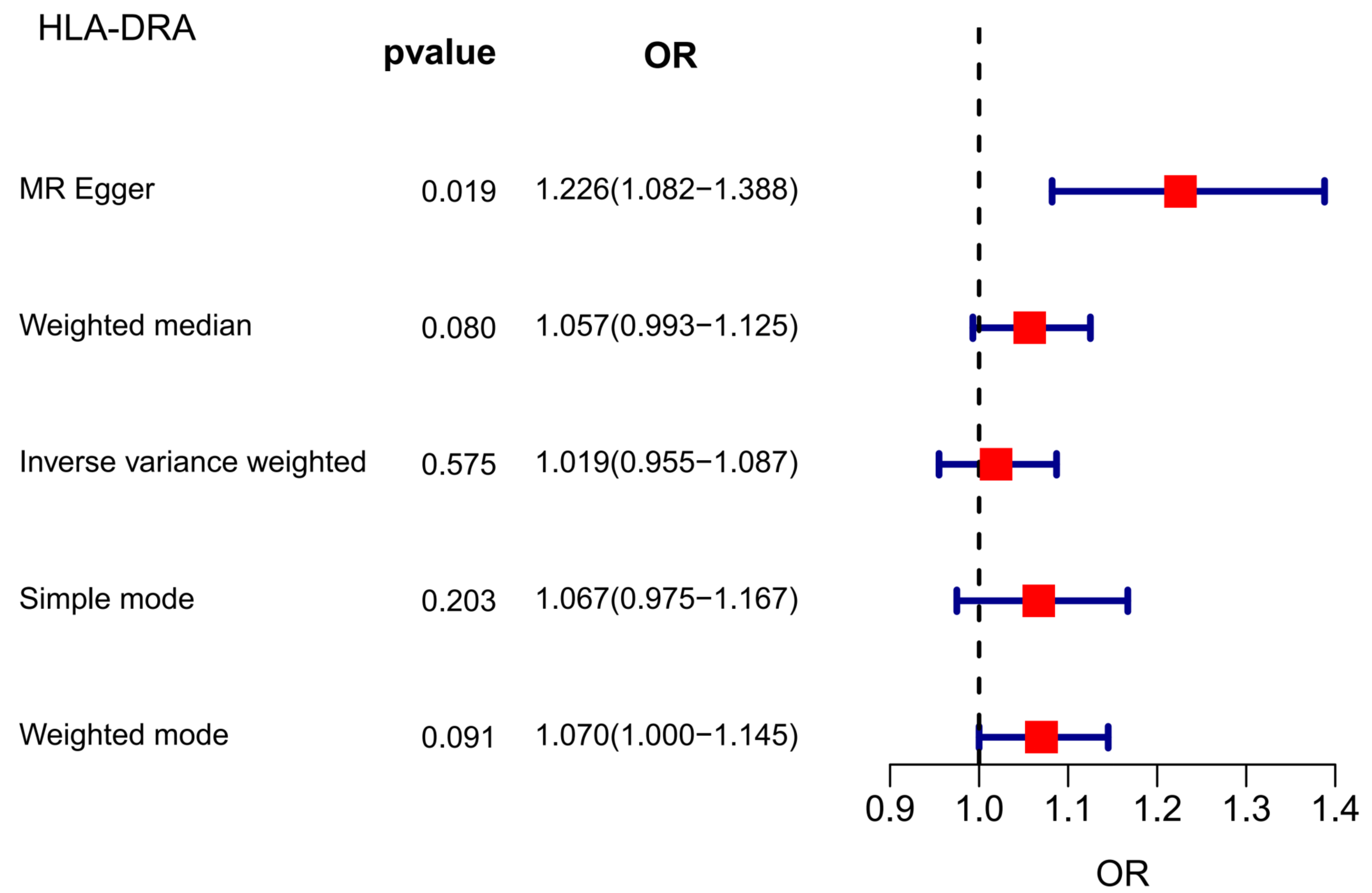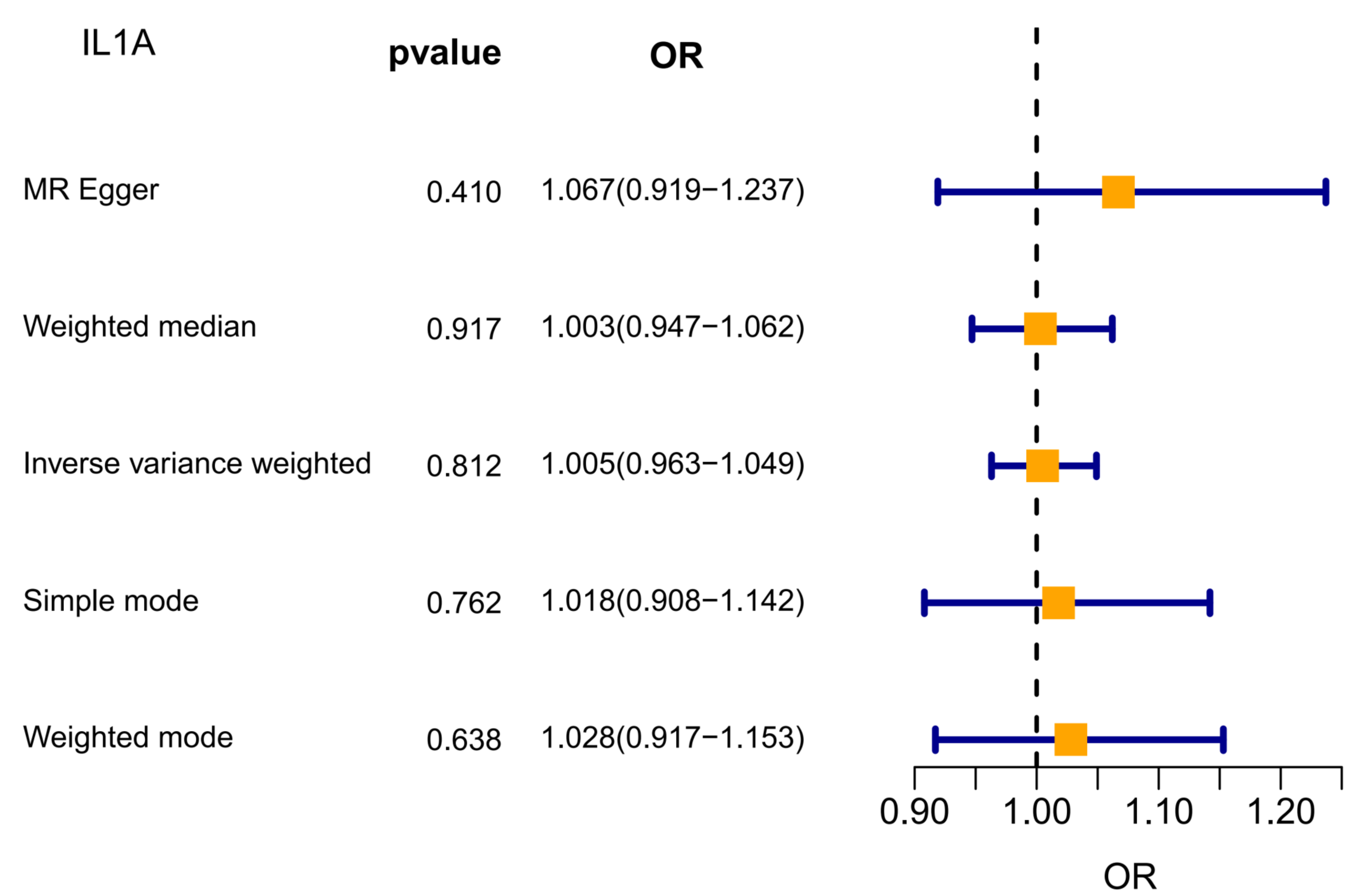

Supplement: Supplementary file 3 — Supporting Information 3 Figure S3: (A) Forest plot of the causal effect of SNP for interleukin‐1α on IS risk. (B) Forest plot of the causal effect of SNP for human leukocyte antigen‐DRα on the risk of IS. [file IJOG-2026-7253270-s003.pdf]
